# Immunomodulatory effects of immune cell-derived extracellular vesicles in melanoma

**DOI:** 10.3389/fimmu.2024.1442573

**Published:** 2024-09-26

**Authors:** Peng Nanru

**Affiliations:** Department of Plastic Surgery, Zhengzhou Central Hospital Affiliated to Zhengzhou University, Zhengzhou, China

**Keywords:** extracellular vesicles (EVs), melanoma, exosomes, T cells, dendritic cells, natural killer cells, macrophages

## Abstract

Melanoma, recognized as one of the most immunogenic malignancies in humans, holds paramount significance in the realm of immunotherapy. However, the emergence of drug resistance and the occurrence of adverse drug reactions underscore the pressing need to explore increasingly personalized immunotherapeutic modalities. Extracellular Vesicles (EVs), pivotal derivatives of immune cells, assume pivotal roles by encapsulating proteins, lipids, and nucleic acids within bilayer lipid structures, thereby facilitating targeted delivery to other immune cells. This orchestrated process orchestrates critical functions including antigen presentation, immune modulation, and the induction of apoptosis in tumor cells. A burgeoning body of evidence underscores the vast therapeutic potential of EVs in melanoma treatment. This comprehensive review aims to delineate the roles of EVs derived from immune cells such as dendritic cells, natural killer cells, macrophages, and T cells in the context of melanoma patients, thereby furnishing invaluable insights for the future direction of melanoma immunotherapy.

## Introduction

Melanoma arises from genetic defects or mutations in melanocytes originating from the neural crest, resulting in a malignant tumor notorious for its high invasiveness compared to other cutaneous malignancies (1, 2). Presently, melanoma incidence is gradually escalating worldwide, with a propensity towards younger onset (3). It is noteworthy that melanoma exhibits the highest somatic mutation rate among human tumors and features widespread expression of neoantigens, concomitant with extensive infiltration of immune cells, rendering it one of the most immunogenic tumors (4). Immunotherapy has progressively emerged as a primary modality for treating melanoma alongside surgical intervention, chemotherapy, and radiotherapy.

The essence of melanoma immunotherapy lies in initiating or augmenting the host’s passive or active immunity against malignant tumors, encompassing immune checkpoint inhibitors (ICIs), cancer vaccines, and adoptive cell transfer (ACT) (5, 6). However, owing to the heterogeneity of melanoma cells across different hosts and diverse evasion mechanisms, the efficacy of immunotherapy manifests significant interindividual variability (7). A comprehensive understanding of the immune system is imperative. Extracellular vesicles have recently garnered attention as a focal point of research, owing to their stable bilayer lipid structure. Initially characterized as a means of selectively eliminating proteins, lipids, and RNA from cells, EVs have gradually unveiled their pivotal role as carriers of intercellular cargo, regulating normal cell homeostasis, and facilitating pathological development (8). Nearly two decades ago, B cells and lymphocytes were found to modulate immune responses through exosome secretion (9, 10). Research on EVs has since expanded to encompass various immune cell types, and their presence in bodily fluids such as blood, urine, saliva, breast milk, amniotic fluid, and ascites has positioned them as novel biomarkers aiding in the diagnosis and prognostication of diverse diseases (11, 12). Herein, we focus on reviewing the roles of immune cell-derived EVs in mediating interactions among immune cells and the potential clinical applications of these derived EVs.

## Generation and sorting of EVs

Based on the mechanisms and sizes of EVs, extracellular vesicles can be categorized into exosomes (with diameters ranging from 30 to 150 nm) and microvesicles (with diameters ranging from 100 nm to 1 μm). Exosomes are primarily secreted via the endosomal pathway, where cargo is transported from the Golgi apparatus to endosomes or internalized from the plasma membrane to form early endosomes (13). During the maturation process of multivesicular bodies (MVBs), intraluminal vesicles (ILVs) bud inward from the endosomal membrane within the MVBs. Eventually, mature MVBs fuse with the plasma membrane, directing exosome secretion into the extracellular space. Conversely, microvesicles are directly budded from the cell membrane outward (14).

It is important to note that while the term “extracellular vesicles” is commonly used to refer to all these secreted membrane vesicles, vesicles derived from different parent cells under different conditions carry various cargoes, leading to significant differences in the fate and function of EVs. This diversity underscores the crucial role of the endosomal sorting complex required for transport (ESCRT) machinery and related accessory proteins (ATPase VPS4, VTA-1, TSG101 and Alix) in cargo sorting (15). Additionally, ceramides and the tetraspanin protein family (CD63, CD81, CD82 and CD9) can also independently participate in cargo sorting via the ESCRT pathway (16).

## Uptake of EVs

**Figure 1:**
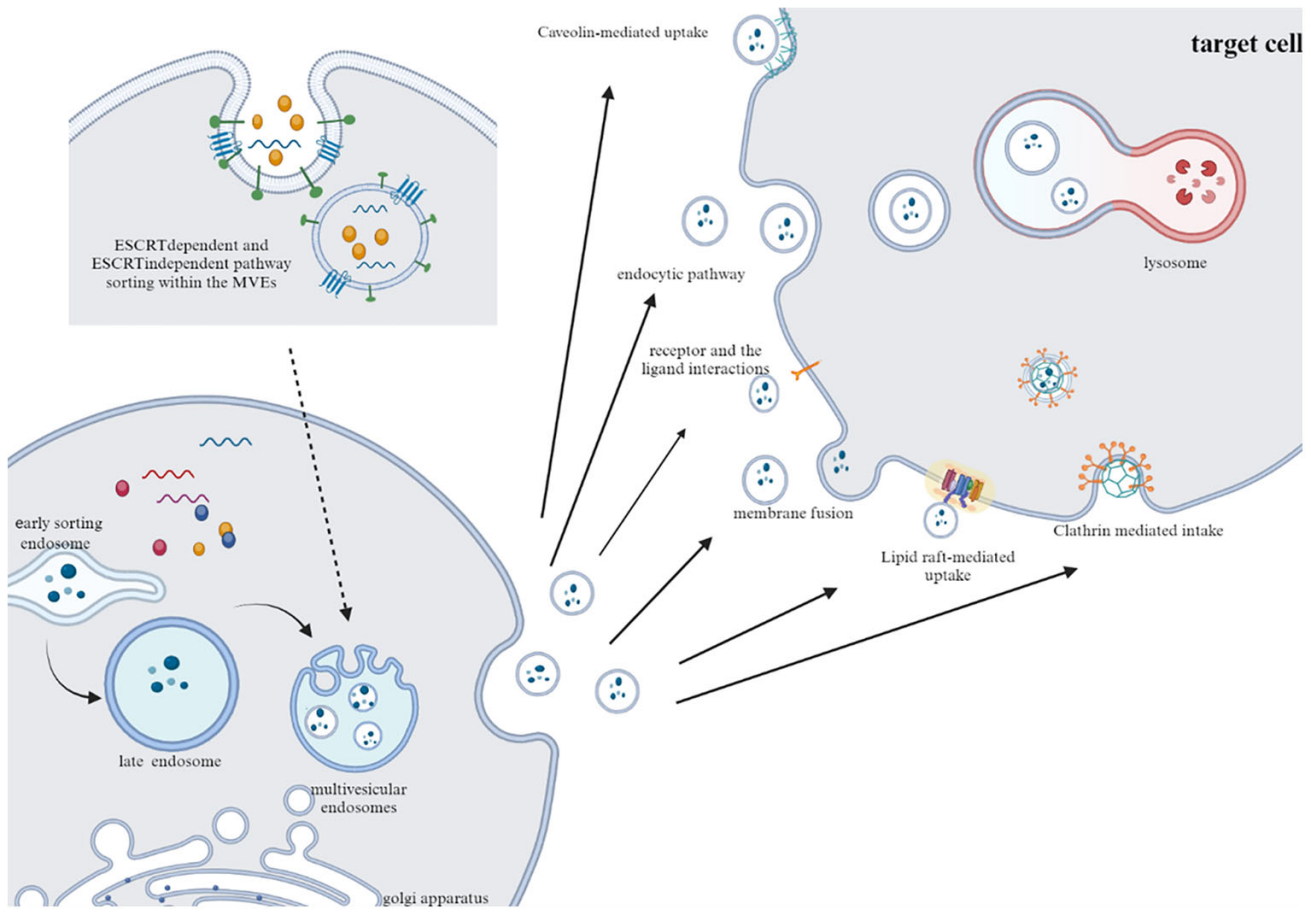
Typical pathways of exosome secretion and uptake. Exosomes are produced by sorting mechanism and endosome pathway. After fusion with cell membrane, exosomes are released to the outside of cells. After transport to target cells, EVs are internalized by cells through phagocytosis, clathrin and caveolin mediated endocytosis.

The internalization of EVs occurs through various endocytic pathways, such as clathrin-mediated uptake, macropinocytosis, phagocytosis, and lipid raft-mediated internalization (17–19). Additionally, tetraspanins, C-type lectin receptors, integrins, T cell receptors (TCRs), and LFA-1 are involved in regulating EVs recruitment and uptake (19). However, it has been observed that EVs internalization is not the sole prerequisite for eliciting phenotypic responses in target cells. EVs can directly induce phenotypic responses through receptor-ligand interactions. Experimental studies by Kerstin Menck and coworkers have demonstrated that the binding of EVs to the surface of target cells can stimulate changes in cellular activity independent of the exchange of EVs contents (20) (**Figure 1**).

## Isolation and recognition of EVs

The acquisition and identification of extracellular vesicles are fundamental for both basic research and clinical applications, yet pose challenges in introducing EVs into clinical practice. Presently, the most common acquisition method is ultracentrifugation (21). Additionally, size exclusion chromatography separates vesicles from other non-vesicular fragments, while immunoaffinity antibody capture selectively isolates vesicles displaying surface markers (22). Ultrafiltration, magnetic immunoprecipitation, and other methods are also employed for EVs isolation (23). Further analysis of EVs involves chemical and physical methods. Chemical analysis includes protein imprinting and flow cytometry, with common markers including tetraspanins (CD9, CD63 and CD81), the protein involved in multivesicular body biogenesis (Tsg101), and cytoskeletal-associated proteins. Physical analysis relies on morphological characteristics of extracellular vesicles, employing techniques such as scanning electron microscopy (SEM), transmission electron microscopy (TEM), dynamic light scattering (DLS), and tunable resistive pulse sensing (TRPS) (24). However, due to the high heterogeneity and diversity of EVs subtypes, there is currently no standardized extraction method.

## Immunomodulatory effect of dendritic cell and its derived EVs in melanoma

Dendritic cells (DCs) serve as highly effective antigen-presenting cells, relying on receptors such as C-type lectin receptors, formyl peptide receptors (FPRs), Toll-like receptors (TLRs), and Fc receptors to mediate phagocytosis or macropinocytosis (25, 26). They recognize internalized antigens and assemble them into specific MHC-peptide complexes. Through direct presentation, cross-presentation, or via derived extracellular vesicles, DCs present antigens to T cells, thereby activating the immune system (25). In melanoma initiation and progression, DCs play a crucial role in coordinating both innate and adaptive immune responses. Additionally, Chen Wang et al. have found that DCs can regulate the tumor antigen-specific CD8+ T cell response in a circadian rhythm-dependent manner, controlled by the differential expression of co-stimulatory molecule CD80 (27). This circadian regulation has shown rhythmic patterns in controlling melanoma volume. DC-based immunotherapy has demonstrated effectiveness in melanoma patients (28, 29).

DC-derived extracellular vesicles (EVs) serve as vital mediators for DC communication, carrying a plethora of membrane proteins (including integrin α and β chains (αMβ2, ICAM-1, MFG-E8, lactadherin, CD1a, b, c and d proteins) to facilitate interaction and fusion with target cells (30). Furthermore, DC-derived small EVs (sEVs) possess the capacity to present antigen peptides derived from parent cells, inducing specific T cell responses, and are capable of accumulating in lymph nodes (31). Studies have demonstrated that compared to parent DCs, DC-derived EVs can transport a greater abundance of antigen peptides, homing specifically to target cells (32).

In experiments conducted by Mungyo Jung et al., it was found that the delivery of tumor antigen-presenting nanovesicles derived from dendritic cells to cytotoxic T lymphocyte-associated antigen 4 (CTLA-4) antibody secreting cells selectively activates tumor-specific T cells. This results in an enhanced anti-tumor efficacy of αCTLA-4 without triggering systemic immune-related adverse events (33). In an immunotherapy study involving postoperative melanoma patients, stimulation of bone marrow DCs with ovalbumin (OVA) antigen modified with CpG ODN led to the delivery of OVA antigen and CpG ODN to tumor-infiltrating dendritic cells (TIDCs) and dendritic cells in tumor-draining lymph nodes (TDLNs) via DC-derived small extracellular vesicles (sEVs). This enhanced DC maturation and anti-tumor immune response effectively overcame immune suppression (34).

DC-derived EVs address the associated drawbacks of DC vaccines, such as cost-effectiveness, stability, and sensitivity to the systemic microenvironment. They have demonstrated safety and good tolerability in phase I and II clinical trials for an increasing number of tumor diseases (35). Additionally, DC-derived EVs serve as transporters. In an experiment conducted by Jiecheng Lin et al., mature dendritic cell-derived exosomes (mDexos) containing BRAF siRNA (siBRAF) targeting mutated BRAF in melanoma were delivered to the melanoma microenvironment via electroporation. This enhanced the serum stability of siBRAF, increased uptake by B16-F10 melanoma cells, and suppressed BRAF gene expression, leading to more effective melanoma treatment (36). In checkpoint-resistant B16 melanoma mouse models, the use of bone marrow-derived dendritic cell-derived EVs loaded with α-galactosylceramide (αGC) and OVA in combination with anti-PD-1 or anti-PD-L1 therapy not only increased CD8+ T cell infiltration but also significantly enhanced the sensitivity of B16 melanoma cells to anti-PD-1 and anti-PD-L1 therapy (37). Furthermore, Anjali Barnwal et al. demonstrated that combined treatment with mDex TA and PLX-3397 in the 16-F10 mouse melanoma model not only increased CD8+T cell infiltration in the tumor microenvironment but also induced a shift from the Th1/Th2 balance to a Th1-dominant phenotype. This regimen depleted tumor-associated macrophages (TAMs) and myeloid-derived suppressor cells (MDSCs) to modulate the TME, reduced FoxP3 expression, and induced a robust systemic anti-tumor immune response in the spleen and lymph nodes (38). These findings highlight the potential of DC-derived EVs to offer new therapeutic strategies for refractory melanoma.

DC-derived extracellular vesicles can activate immune responses through various pathways and exhibit specificity based on different ligand modifications. For instance, dendritic cell-derived exosomes rich in RAE-1γ can specifically activate NK cells and T cells via the NKG2D/NKG2D-L pathway (39). Additionally, in an experiment conducted by Hongmei Cao et al., developed DEV-mimicking aggregation-induced emission (AIE) nanoparticles (DEV-AIE NPs) not only inherited immunomodulatory proteins from parent DCs, thereby activating T cells, but also carried AIE photosensitizer MBPN-TCyP. These photosensitizers selectively accumulated in the mitochondria of tumor cells, inducing advanced immunogenic cell death, thereby achieving synergistic photodynamic and immunotherapy effects to effectively eradicate primary tumors, distant tumors and tumor metastases (40).

DC-derived exosomes are influenced by various factors, and it has been demonstrated that exosomes derived from different maturation stages of DCs exert different effects (41). Additionally, factors such as INF and tumor cell factors also affect the production of DC-EVs. In a recent experiment, it was confirmed that 365 nm near-ultraviolet LED light can promote the efficient production of EVs from mouse bone marrow DC cell line JAWS II cells, and this productivity highly depends on light intensity and exposure time. Furthermore, this experiment also demonstrated that DC-EVs produced under light stimulation exhibit superior quality, high biocompatibility, and immunological functionality (42). DC-derived exosomes also guide the prognosis of melanoma patients. In another experiment, it was confirmed that plasma levels of uPAR+ EVs from both tumor and DC and CD8+T cell sources were higher in melanoma patients who did not respond to immune checkpoint inhibitor immunotherapy. This may represent a new biomarker for innate resistance to immune checkpoint inhibitor immunotherapy (43).

## Immunomodulatory effect of natural killer cells and its derived EVs in melanoma

NK cells serve as frontline immune defense against melanoma, monitoring and controlling its growth and metastatic spread. They primarily kill cancer cells through two cytotoxic pathways: cytolytic granules (perforin, granzymes) and engagement with target cells. They express various molecules including Fas, NKG2D, CD94, perforin, granzymes, CD40L, and others, participating in cytotoxicity, homing, cell adhesion, and immune activation (44). Studies suggest that expansion of highly cytotoxic CD57+NK cells holds promise in adoptive immunotherapy for melanoma (45). Experimental evidence reveals that NK cells upregulate expression of the chemotactic factor HMGB1 upon interaction with melanoma cells, indicating tumor-mediated modulation of NK cell chemokine receptor patterns, thereby enhancing recruitment, movement and patrolling capabilities of NK cells within tumor tissue (46). Additionally, CD56+ NK and NKT cells show promising prognostic potential in metastatic melanoma patients responsive to PD-1 immunotherapy (47).

The exhaustion and dysfunction of NK cells are critical factors contributing to immune suppression and escape in melanoma (48). While melanoma cells have been shown to express various ligands for different NK cell activating receptors, they frequently evade immune therapy by downregulating major histocompatibility complex (MHC) class I molecules, upregulating human leukocyte antigen (HLA), and differentially downregulating expression of NK cell activating immune receptors (NKp30, NKp44, NKp46, NKG2D, DNAM-1), depending on disease stage and anatomical location (49). This impairs their inherent cytolytic activity. Additionally, the functional activity of NK cells can be suppressed by immunosuppressive molecules produced by immune cells and tumor cells in the tumor microenvironment. Experimental evidence by Jan P. Böttcher et al. confirms that tumor-derived prostaglandin E2 (PGE2) partially impairs NK cell viability and inhibits the production of chemotactic factors (CCL5, XCL1 and XCL2), leading to NK cell-cDC1 axis evasion and consequent inhibition of anti-tumor immunity (50). Hypoxia, a characteristic feature of solid tumor development, also diminishes the cytotoxic activity of NK cells (48).

Extracellular vesicles derived from NK cells exhibit constitutive secretion and biological activity. In melanoma patients, both NKExo and NKMV express specific biomarkers such as cytoskeletal proteins, heat shock proteins (HSP60, HSP70), tetraspanins (CD9, CD63, CD81, CD82), and several proteins involved in vesicle transport (Tsg101), synaptic fusion proteins including common markers (CD56, CD63, CD8), and cytotoxic proteins (perforin, granzymes and granulysin) (51, 52). NK-92-Exo tested in a melanoma xenograft mouse model demonstrated significant cytotoxicity against B16F10/effluc cells both *in vitro* and *in vivo* by carrying perforin and FasL functional proteins. Moreover, the abundance of FasL and perforin in NK-92-exo was higher than that in NK cells, and the experiment also showed that NK-92-exo could secrete tumor necrosis factor-α (TNF–α), thus affecting cell proliferation signaling pathways (53). Additionally, experiments conducted by Miriam Aarsund et al. revealed that NK cells or NK-92 cells stimulated with IL-12, IL-15, and IL-18 could more efficiently produce EVs containing cytolytic proteins and promote apoptosis of metastatic melanoma cells with poor responsiveness to NK cell cytotoxicity. This suggests that NK cell-derived EVs can target tumor cells independently of their parent cells. EVs from cytokine-stimulated NK cells also possess advantages in penetrating solid tumors (54).

NK-exo not only exhibits anti-tumor activity *in vitro* and *in vivo*, but also reflects the functionality of NK cells to some extent. In experiments conducted by Cristina Federici et al., ELISA testing of specifically labeled NK-Exo confirmed that the total amount of circulating NKExo in melanoma patients was lower than in healthy donors, with a significant reduction in tsg-101 + CD56 + NKExo. This difference precisely reflects the impairment of NK cell function by tumor cells (52).

## Immunomodulatory effect of macrophages and its derived EVs in melanoma

Tumor-associated macrophages (TAMs), as one of the major infiltrating immune cell types in tumors, can express multiple subtypes under the influence of tumor cells and the tumor microenvironment (55, 56). M1/M2 represent the two extreme states of macrophages, and TAMs can undergo continuous conversion between the M1/M2 paradigms in a dynamic environment. Factors such as IFN-γ, macrophage colony-stimulating factor (M-CSF), CCL2, and CCL5 induce M1-like macrophages (also known as classically activated macrophages), which promote inflammation by producing various pro-inflammatory cytokines (such as IL-12, IL-23) and releasing cytotoxic mediators such as nitric oxide (NO) and reactive oxygen species (ROS), exhibiting effective antigen presentation. Conversely, M2 macrophages inherently possess immunosuppressive properties and can be induced by factors like IL-4, IL-13, IL-10, vitamin D3 and glucocorticoids. They secrete VEGF, IL-8, epidermal growth factor (EGF), platelet-derived growth factor (PDGF), and extracellular matrix (ECM), thereby promoting tumor initiation and metastasis.

In melanoma patients, the infiltration of macrophages often portends a poorer prognosis. Research has shown that melanoma releases high mobility group box 1 protein (HMGB1), a damage-associated molecular pattern, under hypoxic conditions, promoting the accumulation of M2-like TAMs and an intracellular environment rich in IL-10, which suppresses CD8+T cell cytotoxicity and promotes tumor growth (57). Additionally, experiments conducted by Oscar R Colegio et al. indicate that melanoma cells generate lactate via glycolysis under hypoxia, which not only induces the expression of vascular endothelial growth factor (Vegf) and Arg-1 mediated by Hypoxia-inducible factor-1α but also promotes the transformation of macrophages into an M2-like phenotype (58). Furthermore, the mitochondrial and lysosomal states of macrophages themselves can influence their polarization.

Extracellular vesicles derived from tumor-associated macrophages serve as crucial communication tools for exchanging microRNAs, long non-coding RNAs, and proteins with other immune cells and tumor cells, garnering increasing attention in melanoma immunotherapy. Animal cell-level experiments conducted by Kyung-Mi Lee et al. demonstrated that EVs derived from M1 macrophages induced by LPS and INF-γ *in vitro* can promote apoptosis of melanoma cells by upregulating the protein expression of caspase-3 and caspase-7 in melanoma cell lines, while downregulating the expression of immune-inhibitory genes such as Foxp3, CCR4 and CTLA-4, thus facilitating anti-tumor immune responses (59). Furthermore, M1-EVs carrying miR-29a-3p target the transcription factors MAFG and MYBL2 in melanoma cells, inhibiting the transformation of nevi to melanoma and the proliferation of melanoma cells, while downregulating the expression of the oncogene Bmi1, thereby suppressing tumor growth, migration, and invasion (60–62).

Macrophage-derived extracellular vesicles can guide the polarization state of other macrophages and achieve reversal. In one experiment, it was demonstrated that by utilizing exogenously polarized macrophage-derived EVs with similar miRNA content to their parent cells, tumor-associated macrophages treated with M1 nanovesicles were repolarized into M1 macrophages. This “re-education” of TAMs may be associated with differential miRNA expression profiles of M1-EVs (62). In experiments conducted by Gowri Rangaswamy Gunassekaran et al., IL4RPep-1-modified M1-EVs, enhanced with NF-κB p50 siRNA and miR-511-3p, were targeted to the IL-4 receptor of M2 macrophages, resulting in TAM reprogramming towards M1 macrophages. Although the experiment utilized breast cancer cells, IL4R, as a typical immunosuppressive cytokine receptor, is also highly expressed in melanoma patients and closely associated with prognosis (63). However, due to the current focus of TAM-EVs research mainly on cellular and animal levels, this approach only captures part of the tissue microenvironment background, leading to limitations in the study.

## Immunomodulatory effect of CD4+T cells and its derived EVs in melanoma

Mature lymphocytes are primarily classified into CD4+T cells and CD8+ T cells based on their phenotype. Among them, CD4+T cells can be further subdivided into various subtypes, including Th1, Th2, Th3, Th17, Th22, Th9, T regulatory type 1 (Tr1) cells, and CD4 follicular helper T (Tfh) cells, based on their cytokine production and function. They mainly regulate and assist in immune responses (64). With the occurrence of immune escape due to tumor defects in MHC class I molecule expression and exhaustion of CD8+T cells, the cytotoxic role of CD4+T cells has gradually gained attention in melanoma therapy (65). A recent study has confirmed that CD4+ T cells, induced by transcription factors (T-bet, Eomes, runx3, Blimp-1), can differentiate into cytotoxic CD4+ T cells capable of secreting perforin and granzymes, and express protein markers such as CRTAM, CD38, NKG2D, CD26, NKGT, CX3CR1, which are associated with MHC class I-restricted T cell responses (65). In another experiment, tumor-reactive CD4+ T cells transferred into lymphocyte-depleted hosts were shown to expand *in vivo* and directly eliminate established melanomas in an MHC class II-dependent manner (66). Furthermore, experiments conducted by Emma G Bawden et al. have demonstrated that CD4+ T cells not only produce interferon-γ to directly act on melanoma cells but also induce nitric oxide synthase in bone marrow cells to achieve NO-dependent cytotoxicity against melanoma cells (67).

CD4+ T cells also possess the potential to predict the efficacy of melanoma treatment to some extent. Joshua R. Veatch et al. discovered that the local activation of CXCL13+CD4+ T and CD8+ T cells recognizing specific antigens in some melanoma patients, along with the activation of an immune-stimulating phenotype in macrophages and the presence and differentiation of B cells, are associated. They could predict the overall survival of melanoma by calculating the score of CXCL13+CD4+ T cells (68). However, due to the inability of this study to define the antigen specificity of all CD4+ T cells in the CXCL13+ subset and the assumed bystander subset in tumor infiltration, the results have certain limitations.

T cell-derived extracellular vesicles (EVs) play crucial roles in both immune activation and suppression. These vesicles express markers similar to their parent cells and carry constant proteins such as glyceraldehyde 3-phosphate dehydrogenase (GAPDH), enolase, specific heat shock proteins, CD81, CD63, as well as proteins involved in immune processes like human leukocyte antigen I (HLA-I), components of the TCR/CD3 complex, β2-microglobulin, and specific integrins. They achieve phagocytosis through receptor-mediated uptake or formation of immune synapses. EVs from activated CD4+ T cells can activate the STING pathway via IFNγ delivery, thereby reprogramming macrophages to enhance anti-tumor effects (69). Furthermore, Sanghee Shin et al. found that EVs derived from CD4+ T cells transport miR-25-3p, miR-155-5p, miR-215-5p, and miR-375 to CD8+ T cells, inducing proliferation and enhancing the anti-tumor response of CD8+ T cells, without affecting regulatory T cells (Tregs). Interestingly, compared to EVs derived from unstimulated CD4+ T cells, those derived from interleukin-2 (IL-2)-stimulated CD4+ T cells induce a more robust anti-tumor response in CD8+ T cells (70). Similar findings were confirmed in experiments conducted by Dokyung Jung et al., where IL-2-bound Jurkat T cells significantly increased the anti-cancer ability of CD8+ T cells by upregulating the content of miR-181a-3p and miR-223-3p carried by their derived EVs, and downregulating PD-L1 expression in melanoma cells (71).

It is noteworthy that EVs derived from CD4+T cells do not solely exert anti-tumor responses. Haifeng Zhang et al. discovered that extracellular vesicles released by ovalbumin pulsed CD4+T cells can reversely inhibit DC-OVA-mediated CD4+ T cell proliferation and CD8+CTL responses. This may be attributed to the downregulation or masking of pMHC II on DCs by OVA-specific CD4+T cell-derived exosomes or induction of apoptosis in DC-OVA cells expressing Fas via the Fas/FasL pathway (72). The experiment also confirmed that molecular transfer between DCs and T cells is no longer unidirectional but bidirectional.

## Immunomodulatory effect of regulatory T cells and its derived EVs in melanoma

Regulatory T cell (Tregs), as a subtype of CD4+ T cells, play a crucial role in maintaining immune homeostasis in normal conditions. However, in melanoma patients, they inhibit activated cytotoxic T cells by expressing various immune checkpoints (CTLA4, PD1, Tim-3, LAG-3) and secreting inhibitory cytokines (TGF-β/TGF-b, IL-10, IL-35) to interact with other immune cells (73). Additionally, they can activate tumor microenvironment TGFβ by producing αvβ8 integrin (Itgβ8), promoting the development of an immunosuppressive microenvironment (74). Yufeng Xie et al. found that EVs derived from natural CD8+CD25+ regulatory T cells significantly inhibit DC-induced cytotoxic T lymphocyte responses and anti-tumor immunity in a mouse B16 melanoma model (75). Sim L Tung et al. confirmed that Tregs can also modulate other immune cells by transferring miR-150-5p and miR-142-3p via EVs, thereby regulating cytokine release and inhibiting phagocytosis (76). Furthermore, in another experiment, it was found that Treg-derived exosomes can promote their inhibitory function by converting extracellular 5’-AMP to adenosine through CD73 expression. Adenosine interacts with adenosine receptors expressed on effector T cells, triggering cAMP production inside the effector T cells, thus inhibiting cytokine production. However, further evidence is needed to demonstrate whether this potential immunosuppressive mechanism occurs *in vivo (* 77).

## Immunomodulatory effect of CD8+ T cells and its derived EVs in melanoma

CD8+T cells, as the most important effector cells among cytotoxic T cells, can specifically recognize and kill cancer cells. It is believed that CD8+T cells mainly induce cancer cell death through perforin and granzymes, as well as Fas-L/Fas binding. In recent years, research has found that CD8+T cells can also exert anti-tumor effects by promoting ferroptosis in cancer cells through the secretion of IFN-γ (78).

The process of the immune system eliminating tumors is not smooth. Although the immune escape mechanisms of melanoma are not yet clear, current research has confirmed that defects in antigen processing and presentation pathways, downregulation of MHC class I molecules, and increased expression of immune checkpoint molecules (PD-1/CTLA-4, TIM-3, LAG 3 and VISTA) can lead to abnormal antigen presentation, exhaustion of effector cells, and protection of tumor cells from attack (79). Additionally, the expression of certain transcription factors (ZEB1, PCSK9, HIF-1, AP-1 and NF-κB) and related enzymes (HSD11B1, ALDH2) also participate in driving tumor immune evasion (80).

Interestingly, the immune response mediated by CD8+T cell-2derived vesicles is largely dependent on the state of the parent cells and participates in various immune escape mechanisms. Research has shown that activated CD8+T cells secrete FasL+ exosomes, which can accelerate invasion of melanoma cells *in vivo* by activating ERK and NF-κB pathways and upregulating MMP9 expression (81).Additionally, studies by Xiaochen Wang et al. have confirmed that functional CD8+T cells can uptake exosomes derived from damaged CD8+T cells, leading to inhibition of normal CD8+ T cell proliferation, cell activity, and interferon-γ and interleukin-2 cytokine production through the transfer of lncRNA (82). Furthermore, experiments by Yufeng Xie et al. have also demonstrated that exosomes derived from activated CD8+ T cells can induce apoptosis of dendritic cells and suppress the anti-tumor activity of CD8+ T cells in melanoma models by downregulating MHC-I and Fas/FasL pathways in DCs (83). However, Naohiro Seo et al. found that activated CD8+T cells from healthy mice, rather than chronically exhausted CD8+T cells in tumor-bearing mice, release extracellular vesicles carrying cytotoxic miRNA (miR-298-5p). These vesicles induce apoptosis of stromal tumor cells through the surface expression of death ligands such as FasL, TNF-α, and PD-L1, leading to significant attenuation of tumor invasion and metastasis (84).

**Figure 2:**
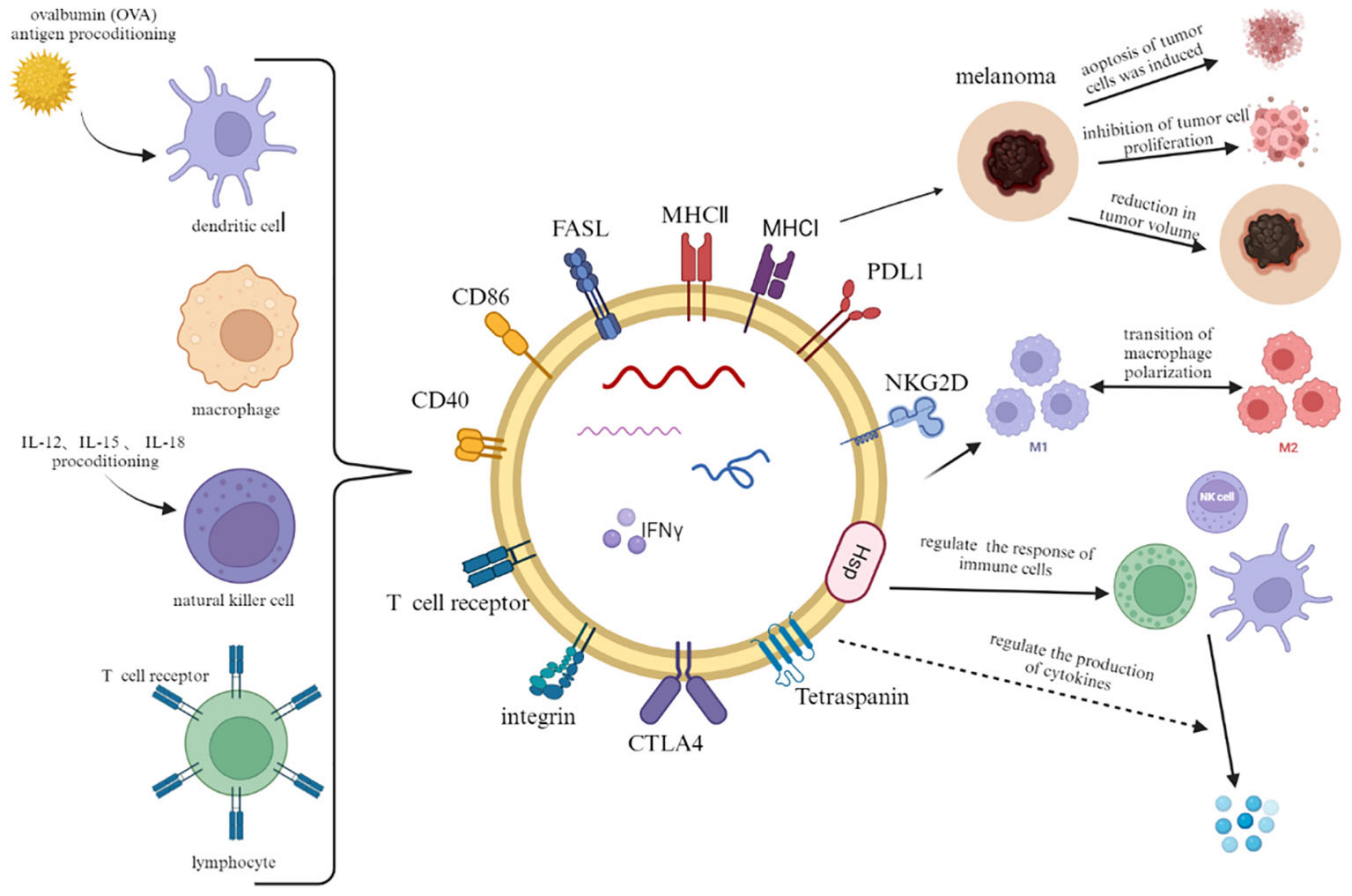
In addition to a part of constant structural proteins, immune cell-derived extracellular vesicles also carry plasma membrane proteins, RNA and lipids from parental cells. When combined with melanoma cells, they can induce tumor cell lysis, inhibit proliferation and reduce tumor volume. In addition, they crosstalk with other immune cells to regulate immune response and cytokine production.

Extracellular vesicles derived from adaptive immune cells not only exert immunosuppressive and immunostimulatory effects but also hold great potential in predicting the efficacy of immunotherapy. Simona Serratì et al. demonstrated through experiments that high levels of PD1+ EVs from T cells and B cells, as well as high levels of PD-L1+ EVs from melanoma cells, serve as independent response biomarkers (85). In the study by Porcelli et al., it was found that melanoma patients resistant to immune checkpoint inhibitors had lower levels of uPAR+ EVs derived from CD8+ T cells in their blood, which could serve as an important predictor of immune therapy with checkpoint inhibitors in metastatic melanoma (43) (**Figure 2**).

## Applications of immune cell-derived EVs in melanoma

Melanoma is one of the most immunogenic tumors, making immunotherapy significant in its treatment. The goal of immunotherapy is to modulate the host’s immune response to the tumor. Common immunotherapy approaches include exogenous supplementation of cytokines such as interleukin-2 (IL-2) and interferon-alpha (IFN-α), active and adoptive immunotherapy, targeted immune checkpoint inhibition, and vaccination (7, 86). However, with the emergence of resistance and side effects during treatment, there is a demand for safer and more effective therapies. Immunocyte-derived EVs have garnered attention in cancer immunotherapy due to their immunogenicity and molecular transfer capabilities. They can utilize surface molecules such as antibodies, proteins, peptides, or small molecules to actively target receptors expressed or overexpressed in cancer cells or the tumor microenvironment, thereby enhancing therapeutic efficacy, reducing side effects, and minimizing immunogenicity. Clinical trials have demonstrated the safety and scalability of Exo vaccines, which carry tumor-associated antigen (TAA) subunits, in patients with advanced melanoma).

In addition, the intrinsic and modified properties of EVs obtained through nanotechnology offer promising prospects for melanoma treatment. Loading the BRAF V600E mutation-specific drug (PLX4032) into BPLP-PLA nanoparticles results in the internalization of nanoparticles within macrophages, which further deliver the drug to melanoma cells through cell-cell binding, thus minimizing the toxicity of cancer drugs to immune cells and other healthy cells (87). Furthermore, studies have shown that PEG-PLGA nanoparticles coated with neutrophil membranes loaded with rapamycin exhibit significantly enhanced cytotoxicity and apoptosis rates in mouse melanoma cell line B16F10 (88). In another experiment, it was found that macrophage-derived extracellular vesicles carrying AO are more efficiently taken up by melanoma cells compared to free AO, enhancing AO’s tumor-killing effect by increasing the exposure time to biological targets (89). Antonella Barone et al. achieved a hybrid nano system by fusing M1 macrophage-derived extracellular vesicles (EVs-M1) with heat-responsive liposomes, resulting in a synergistic effect between the characteristics of liposomes and EVs-M1, thereby enhancing the therapeutic efficacy of the payload and achieving higher tumor accumulation (90). In a recent study, DNA hydrogels loaded with natural killer cell-derived extracellular vesicles exhibited enhanced efficacy in synergistic immunotherapy and photodynamic therapy, inhibiting tumor growth (91). It is evident that both naturally derived immune cell-derived EVs and engineered immune cell-derived EVs hold tremendous potential in the treatment of melanoma patients.

## Conclusion

Immunotherapy holds significant importance in the treatment of melanoma patients, serving not only as adjuvant therapy after surgical resection but also as treatment for advanced (unresectable or metastatic) stages. However, existing immunotherapies have certain limitations, leading to increasing attention on the development of novel immunotherapeutic approaches in melanoma research. With the growing understanding of extracellular vesicles (EVs), emerging data suggest that immune cell-derived EVs possess remarkable abilities to modulate the tumor microenvironment immune response. Immune cell-derived EVs regulate the differentiation, maturation, proliferation, and function of other immune cells, including dendritic cells (DCs), CD8+ T cells, CD4+ T cells, macrophages, and NK cells, to control the progression of melanoma. Moreover, owing to their specific homing ability and stable double-layered lipid structure, EVs have become promising tools for drug delivery and carriers of tumor antigens. By reviewing relevant studies, a more comprehensive understanding of the role of immune cell-derived EVs in melanoma development is provided. However, it is imperative to overcome the need for more accurate methods of EVs extraction and identification, as failure to do so may impede the application of EVs in melanoma therapy. Our review offers insights into the potential mechanisms of immune cell-derived EVs in treating melanoma and provides insights into future research directions. In the future, more work is required to better understand the EVs mechanism of action and further advance its application in these melanoma immunotherapy fields.
